# Generation of Murine Sympathoadrenergic Progenitor-Like Cells from Embryonic Stem Cells and Postnatal Adrenal Glands

**DOI:** 10.1371/journal.pone.0064454

**Published:** 2013-05-10

**Authors:** Shobhit Saxena, Joachim Wahl, Markus S. Huber-Lang, Dominic Stadel, Peter Braubach, Klaus-Michael Debatin, Christian Beltinger

**Affiliations:** 1 Department of Pediatrics and Adolescent Medicine, University Medical Center Ulm, Ulm, Germany; 2 Institute of Traumatology, Hand- and Reconstructive Surgery, Ulm University, Ulm, Germany; 3 Division of Neurophysiology, Ulm University, Ulm, Germany; National Institutes of Health, United States of America

## Abstract

Sympathoadrenergic progenitor cells (SAPs) of the peripheral nervous system (PNS) are important for normal development of the sympathetic PNS and for the genesis of neuroblastoma, the most common and often lethal extracranial solid tumor in childhood. However, it remains difficult to isolate sufficient numbers of SAPs for investigations. We therefore set out to improve generation of SAPs by using two complementary approaches, differentiation from murine embryonic stem cells (ESCs) and isolation from postnatal murine adrenal glands. We provide evidence that selecting for GD2 expression enriches for ESC-derived SAP-like cells and that proliferating SAP-like cells can be isolated from postnatal adrenal glands of mice. These advances may facilitate investigations about the development and malignant transformation of the sympathetic PNS.

## Introduction

Peripheral sympathoadrenergic cells develop from neural crest cells. Signals emanating from surrounding cells such as the BMPs (bone morphogenetic proteins), FGF (fibroblast growth factor) and Wnts (wingless-type proteins) induce neural crest markers including SNAIL/SLUG (vertebrate homologs of *Drosophila* snail gene), PAX3 (paired box 3), SOX9/10 (sex determining region Y-box) [1]. Migratory neural crest stem cells (NCSCs) express CD57 (HNK-1) and MYCN [2], [3]. Once in the proximity of the dorsal aorta, BMPs induce a network of transcription factors in NCSCs that specify them to become sympathoadrenergic progenitors (SAPs) [4]–[6]. Within this network PHOX2b (paired-like homeobox 2b) is pivotal and MASH1 (mammalian achaete schute homolog 1) is important [7], [8] **.** These transcription factors induce HAND2 (heart- and neural crest derivatives-expressed protein 2) and GATA3 (GATA binding protein 3), which in concert with PHOX2b induce key enzymes of catecholamine biosynthesis, TH (tyrosine hydroxylase) and DBH (dopamine beta-hydroxylase) [9]–[11]. Additional factors then differentiate SAPs towards mature sympathetic neurons and chromaffin cells. *In vitro*, those factors include glial cell line-derived nerve growth factor (GDNF), neurturin (NTN), brain-derived neurotrophic factor (BDNF), neurotrophin 4 (NT4), activating tyrosine kinase receptor B (TRKB), nerve growth factor (NGF), neurotrophin 3 (NT3) and activating tyrosine kinase receptor A (TRKA) [12], [13].

An important pathology of the sympathetic peripheral nervous system is neuroblastoma (NB), where amplification of MYCN is strongly associated with poor prognosis [14]. The c-myc-immortalized neural crest progenitor-like cell line JoMa1 can be transformed to neuroblastoma-like cells upon substitution of its c-myc activity by MYCN [15]. Transgenic mice overexpressing human MYCN under the control of the rat TH promoter develop NB [16]. Data from this mouse model point to SAPs as putative cells of origin of NB [16]–[19], a notion supported by recent findings of cooperation between MYCN and NB-associated ALK mutant in chick SAPs [20], zebra fish [21], and mice [22].

Experimental access to NCSCs and SAPs is crucial for investigating normal as well as aberrant development of the sympathetic PNS. However, derivation of NCSCs and SAPs from ES cells and peripheral tissue is complicated by cellular heterogeneity and low yield. We therefore set out to improve isolation of SAPs. In this paper we provide evidence that selecting for expression of GD2 enriches for ESC-derived SAP-like cells and that proliferating SAP-like cells can be isolated from postnatal adrenal glands.

## Materials and Methods

### Ethics Statement

The animal studies were approved by the Regierungspräsidium Tübingen (permit number 986). All animal experiments were carried out in strict accordance with institutional and state guidelines for animal welfare and all efforts were made to minimize suffering.

### Mouse Embryonic Stem Cell Culture

The murine embryonic stem cell (mESC) line D3 was cultivated on mitotically inactivated primary mouse embryonic fibroblasts (MEFs) as described elsewhere [23].

### Generation of ESC-derived Neural Progenitor Cells (NPCs)

Embryoid bodies (EBs) generated from mESCs by the hanging drop method [23] were plated in Iscove’s Modified Dulbecco’s Medium (Life Technologies, Darmstadt, Germany) containing 10% FCS (Life Technologies) on 0.1% gelatin-coated dishes. After 1 d, medium was replaced with Dulbecco's Modified Eagle Medium:Ham’s F-12 (DMEM/F-12, Life Technologies) supplemented with 5 µg/ml insulin, 50 µg/ml transferrin, 30 nM selenium (all from Sigma-Aldrich, Munich, Germany) and 5 µg/ml fibronectin (Life Technologies). Cells were cultured at 37°C and 5% CO_2_ for 7 d, generating NPCs.

### Low-level Enrichment of NCSCs from NPCs

Dissociated NPCs were plated on poly-D-lysine/fibronectin (both at 150 µg/ml, Sigma-Aldrich) coated culture dishes and cultured in NCSC medium, consisting of 5∶3 DMEM low-glucose:neurobasal medium (Life Technologies) supplemented with 20 ng/ml bFGF (Miltenyi Biotec, Bergisch Gladbach, Germany), 20 ng/ml IGF-1 (R&D Systems, Minneapolis, USA), 1% N2 supplement, 2% B27 supplement (both from Life Technologies), 35 ng/ml retinoic acid (Sigma-Aldrich), 50 µM β-mercaptoethanol (Serva, Heidelberg, Germany) and 15% chicken embryonic extract [24]. The cells were cultured for 7 d in a hypoxia chamber (BioSpherix, Lacona, USA) adjusted to 3% O_2_.

### Generation of Higher-enriched NCSC-like Cells by Sorting for CD57 Expression

FACS sorting of low-enriched NCSC-like population for expression of CD57 generated higher-enriched NCSC-like cells. Briefly, cells were stained with CD57 antibody (clone NK1, Abcam, Cambridge, UK) at a concentration of 0.1 µg/1×10^6^ cells for 30 min on ice, followed by three washes with PBS (phosphate buffered saline). Cells were sorted with a FACSAria cell sorter (BD Biosciences, Heidelberg, Germany). Dead cells were marked by SYTOX Blue nucleic acid stain (Life Technologies) and CD57^−^ and CD57^+^ fractions were sorted from live cells.

### Higher Enrichment of SAP-like Cells by Sorting for Expression of GD2

Low-enriched NCSC-like population were FACS-sorted for GD2 expression to generate highly-enriched SAP-like cells. GD2 antibody (Clone 14.G2a, BD Biosciences) was used to stain the cells (0.5 µg/1×10^6^ cells) for 30 min, followed by FACS sorting as described above.

### Isolation and Culture of Adrenal-derived Spheres

C57BL/6 mice (2 d old) were sacrificed and the adrenal glands were removed under sterile conditions. Glands were incubated with Liberase Blendzyme solution (0.62 U/ml, Roche Diagnostics, Mannheim, Germany) for 45 min at 37°C. After mechanically dissociating the tissue and neutralizing the enzyme with 10% FCS cells were passed through a 70 µm cell strainer (BD Biosciences) and seeded at a density of 1.2×10^5^ cells/ml in serum-free sphere medium consisting of DMEM/F-12 medium supplemented with bFGF and EGF (20 ng/ml each; Miltenyi Biotec), 20 ng/ml LIF (Millipore, Schwalbach, Germany), 10 U/ml heparin (Ratiopharm, Ulm, Germany) and 2% B27 supplement. Cells were cultured in low-attachment plastic dishes at 37°C and 5% CO_2_. Differential plating was employed [25], where plated cells were transferred after 2 h, 8 h and 3 d to a new culture dish in order to eliminate adrenocortical and endothelial cells. For immunocytology, spheres were plated overnight on poly-D-lysine/fibronectin-coated glass coverslips in sphere medium containing 1% FCS.

### Flow Cytometry

To analyze surface proteins, cells were directly labeled with primary antibodies (or appropriate isotype control) for 30 min. To detect intracellular proteins, cells, fixed with 1% paraformaldehyde were permeabilized with 0.3% IGEPAL (Sigma-Aldrich), blocked with 3% BSA, and incubated with primary antibodies (Table S1) in blocking buffer. After applying appropriate secondary antibodies, cells were washed and 1×10^4^ events were acquired with BD FACScan; data was analyzed using CellQuest (both from BD Biosciences).

### Immunocytology

Cells were fixed with cold ethanol for 10 min at −20°C, permeabilized for 5 min with 0.3% IGEPAL, blocked for 30 min with 4% goat serum and 0.4% BSA in PBS and incubated overnight at 4°C with primary antibodies (Table S1). For detection, cells were incubated with fluorochrome-conjugated secondary antibodies (Table S1) for 30 min. To detect horseradish peroxidase (HRP)-conjugated secondary antibodies or EnVision™^+^ Dual Link polymer HRP (Dako, Denmark), diaminobenzidine solution (Sigma-Aldrich) was added for 10 min at room temperature. After counterstaining the nuclei with 10 µg/ml 4′, 6-diamidino-2-phenylindole dihydrochloride (DAPI, Sigma-Aldrich) and mounting in mounting medium (Dako, Glostrup, Denmark), cells were viewed using Olympus Provis microscope (Olympus, Hamburg, Germany). Images were acquired using analySIS (Soft Imaging System, Münster, Germany).

### Reverse Transcription PCR

Total RNA was isolated using TRIzol® reagent (Life Technologies). cDNA was synthesized using SuperScript® III First-Strand synthesis system (Life Technologies). PCR was carried out with 200 nM dNTPs and 0.1 U/µl Taq DNA Polymerase (both from Sigma-Aldrich). Primers spanned exon-intron boundaries (primer sequences in Table S2). PCR consisted of an initial denaturation at 94°C for 4 min followed by 29 cycles of denaturation at 94°C for 30 s, annealing for 30 s, and extension at 72°C for 45 s.

### Quantitative Real Time PCR

Real-time PCR was carried out using a LightCycler (Roche Diagnostics). 2.5 µl cDNA was amplified with 1x reaction mix (LightCycler FastStart DNA Master, Roche) that included SYBR Green I, 10 pmole of each primer and 2.5 mM MgCl_2_ in a final volume of 10 µl. Cycling conditions were 95°C for 10 min, followed by 45 cycles with 95°C for 10 s, annealing for 5 s, and 72°C for 10 s. After the final cycle melting curve analysis was performed to confirm correct product amplification. The relative expression (R) of marker gene mRNA compared to TATA-box-binding protein 1 (TBP1) mRNA was calculated as: R = E×TBP1 ^CP TBP1^/E×marker gene ^CP marker gene^, with E representing the RT-PCR efficiency and CP the crossing points.

### BrdU Assay

5-bromo-2′-deoxy-uridine (BrdU) labeling and Detection Kit (Roche) was used. Adrenal-derived spheres were treated with 10 µM BrdU labeling reagent for 24 h. BrdU-treated spheres, dissociated to single cells, were plated on poly-D-lysine/fibronectin coated coverslips for 3 h and were stained with anti-BrdU antibody as per the manufacturer’s instructions.

### Electron Microscopy

Adrenal-derived spheres were fixed with 2.5% glutaraldehyde in 1% sucrose containing 0.1 M phosphate buffer (pH 7.3) and were post fixed in 2% osmium tetroxide for 2 h. After dehydrating through a series of graded ethanol to propylene oxide, the sample was embedded and polymerized in epoxy resin. Sections (0.5 µm) were examined with a Zeiss EM-10 electron microscope (Carl Zeiss, Oberkochen, Germany).

### Electrophysiology

Adrenal-derived spheres were incubated with 10 µM of a potentiometric indicator dye di-8-amino-naphthylethenylpyridinium (di-8-ANEPPS) at 37°C for 30 min, followed by washing with Krebs-Ringer solution. Emission was filtered at 501 nm and detected by a photomultiplier. Action potentials were triggered by rectangular pulses (10 V, 0.5 ms) through two stainless steel electrodes in the bath solution directly adjacent to the cells of interest. Double pulses at decreasing intervals were used to determine the refractory period of the compound action potentials (CAP). Four traces were averaged and filtered with a 101 point central rolling mean. Baseline correction and bleaching correction were performed before normalizing to the peak of the initial CAP using macros written in R [26]. For whole-cell voltage-clamp recordings (Axopatch-200B, Axon instruments, Berkeley, CA, USA), the bath solution contained (in mM) 150 NaCl, 2 KCl, 1.5 CaCl_2_, 1 MgCl_2_, 10 HEPES (pH 7.4; 291 mOsm), patch pipette (tip resistance 2.5–4 MΩ) contained (in mM) 105 CsF, 35 NaCl, 10 EGTA, 10 HEPES (pH 7.4; 287 mOsm). Pipette resistances were 2.5–4 MΩ and seal resistances 1–5 GΩ.

### *In vitro* Differentiation

For differentiation of GD2-sorted NCSC-derived SAP-like cells towards chromaffin lineage, GD2^+^ cells were differentiated for 6 d on poly-D-lysine/fibronectin coated coverslips in NCSC medium supplemented with 10 µM dexamethasone (Sigma-Aldrich) and 100 nM Phorbol 12-myristate 13-acetate (PMA, Millipore). For differentiation of adrenal-derived spheres, basal differentiation media consisted of DMEM/F-12 supplemented with 1% B27, 30 mM glucose (Sigma-Aldrich), 1 mM glutamine and 50 ng/ml BSA (Sigma-Aldrich). Spheres were differentiated in adherence on poly-D-lysine/fibronectin-coated coverslips for 6 d in this differentiation media supplemented with a combination of 10 µM all-trans retinoic acid (ATRA, Sigma-Aldrich) and 100 µM ascorbic acid (Sigma-Aldrich) for neural differentiation and a combination of 10 µM dexamethasone and 100 nM PMA for chromaffin differentiation.

### Intra-adrenal Orthotopic Transplantation

Dissociated cells of spheres derived from the adrenal glands of 2 d old mice were labeled with 5 µM CFSE (carboxyfluorescein succinimidyl ester, Life Technologies) according to the manufacturer’s instructions. The labeled cells were resuspended in saline containing fibrinogen (8 mg/ml, Sigma-Aldrich). Thrombin (8 U/ml, Sigma-Aldrich) was added to this cell suspension to induce clotting. Using a retroperitoneal approach, clots containing 5×10^5^ cells were microsurgically positioned via a 2 mm incision within the adrenal glands of 8–12 week old nude rats (Charles River, Sulzfeld, Germany) and closed with a 9–0 suture.

### Immunohistochemistry

Rat adrenal glands were frozen in Tissue-Tek® O.C.T.™ (Sakura Finetek, Torrance, CA) in stainless-steel molds chilled on liquid nitrogen. Adrenal gland cryosections (3 µm) were fixed with 4% paraformaldehyde for 15 min, permeabilized for 5 min with 0.3% IGEPAL, blocked for 30 min with 4% goat serum/0.4% BSA in PBS and incubated overnight at 4°C with primary antibodies (Table S1). After three washes with PBS, appropriate horseradish peroxidase-conjugated secondary antibodies or EnVision™^+^ Dual Link polymer-HRP (for nestin detection) were applied (Table S1). Detection was carried out with diaminobenzidine solution (Sigma-Aldrich) and after three washes with PBS, the sections were mounted in DAKO mounting medium for microscopic observation.

### High-performance Liquid Chromatography for Catecholamine Detection

Cells were homogenized in 250 µl sonication buffer consisting of 0.4 M perchloric acid, 0.5 mM sodium metabisulfite and 2% EDTA (all from Sigma-Aldrich). Qsonica sonicator (Newtown, CT, USA) was used to sonicate the cells on ice with 3 bursts (5 s each) at 50% amplitude, at 25 s intervals. Sonicated lysate was centrifuged for 5 min at 10000 rpm and the supernatant was used to analyze catecholamines by high performance liquid chromatography-electrochemical detection (HPLC-ECD). Internal standard used with each run was 150 pg 3,4-dihydroxybenzylamine (Chromsystems, Gräfelfing, Germany). After neutralization with 6 ml neutralization buffer (Chromsystems), the solid-phase extraction column (Chromsystems) was filled with the sample and eluate was discarded. The column was washed with HPLC-grade water (Merck, Darmstadt, Germany). Substances were eluted with 6 ml elution buffer (Chromsystems). 5 M Hydrochloric acid (30 µl, Chromsystems) was added per 1 ml eluate and 20 µl of the acidified eluate was used to separate and quantify catecholamines by HPLC-ECD using Agilent 1100 series HPLC system (Agilent Technologies, Böblingen, Germany) and electrochemical detector EC3000 (Recipe Chemicals, Munich, Germany).

### Statistical Analysis

Statistical analysis was performed using GraphPad Prism (GraphPad Software, San Diego, CA, USA). For statistical evaluation, student’s t-test was used and results were considered to be statistically significant at p-value < 0.05.

## Results

### NPCs Differentiated from ES Cells Contain Cells Expressing Markers of NCSCs and SAPs

**Figure 1:**
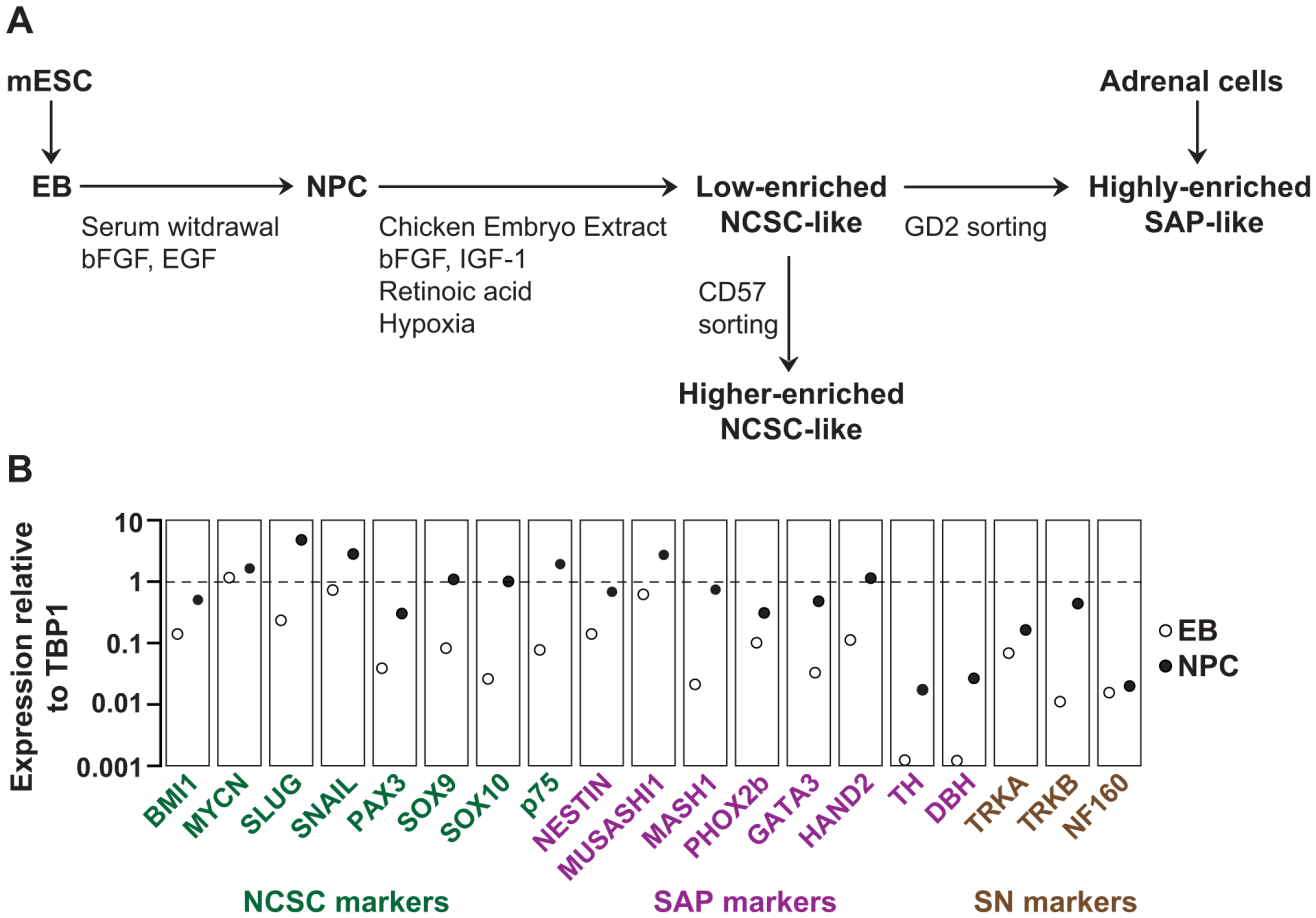
Strategies and selection of neural progenitor cells. (**A**) **Strategies to generate NCSCs and SAPs.** Two strategies were used, derivation of NCSC-like cells and SAP-like cells from murine embryonic stem cells (ESCs) and isolation of SAP-like cells from postnatal adrenal glands of mice. For derivation from ESCs, germ layer specification was induced by forming embryoid bodies (EBs) from mouse embryonic stem cells (mESCs). NPCs were differentiated from EBs by serum deprivation and mitogen addition. By applying culture conditions supportive for neural crest stem cells, a low-enriched NCSC-like population was generated. Sorting this population for CD57^+^ cells led to a population of higher-enriched NCSC-like cells while sorting for GD2^+^ cells generated a fraction of highly-enriched SAP-like cells. For isolation of SAP-like cells from postnatal adrenal glands, dissociated adrenal cells are grown as spheres in serum-free medium. (**B**) **NPC-lineage selection from ESCs generates cells expressing markers of NCSCs and SAPs.** EBs generated from murine ES cells were subjected to NPC-lineage selection. Specific mRNA levels of genes associated with NCSCs, SAPs and sympathetic neurons were analyzed by qRT-PCR and are shown in relation to TATA-Box binding protein-1 (TBP1). RNA was isolated on d 4 of EB differentiation and d 7 of NPC culture. The experiment was repeated twice with similar results.

A widely used strategy to generate neural progenitor cells (NPCs) from ESCs involves the formation of embryoid bodies (EBs), followed by culture in serum-free medium with mitogens to induce and expand NPCs [27], [28] (Fig. 1A). We investigated in detail whether differentiation of ES cells to NPCs cells also yields cells expressing markers related to NCSCs and SAPs. To this end, we compared EBs and NPCs by RT-PCR for their relative mRNA levels of genes characteristically expressed in NCSCs, SAPs and maturing sympathetic neurons. After differentiating EBs in serum-free medium, expression of NCSC markers increased in the NPC population, i.e. BMI1, MYCN, SLUG, SNAIL, PAX3, SOX9, SOX10 and the low-affinity NGF-receptor p75 (Fig. 1B). SAP markers were also upregulated in NPCs, notably nestin, MUSASHI1, MASH1, PHOX2b, GATA3, HAND2, TH and DBH. Expression of neurotrophin receptors associated with survival of maturing sympathetic neurons also increased, i.e. TRKA and TRKB, as did the expression of NF160 (Fig. 1B).

These data show that by using conditions for NPC selection, cells are generated expressing markers characteristic for NCSCs and SAPs, and – less pronounced – markers associated with maturing sympathetic neurons.

### Adaption of Culture Conditions and Sorting for CD57 Expression Enriches for ESC-derived NCSC-like Cells but not for SAP-like Cells

**Figure 2:**
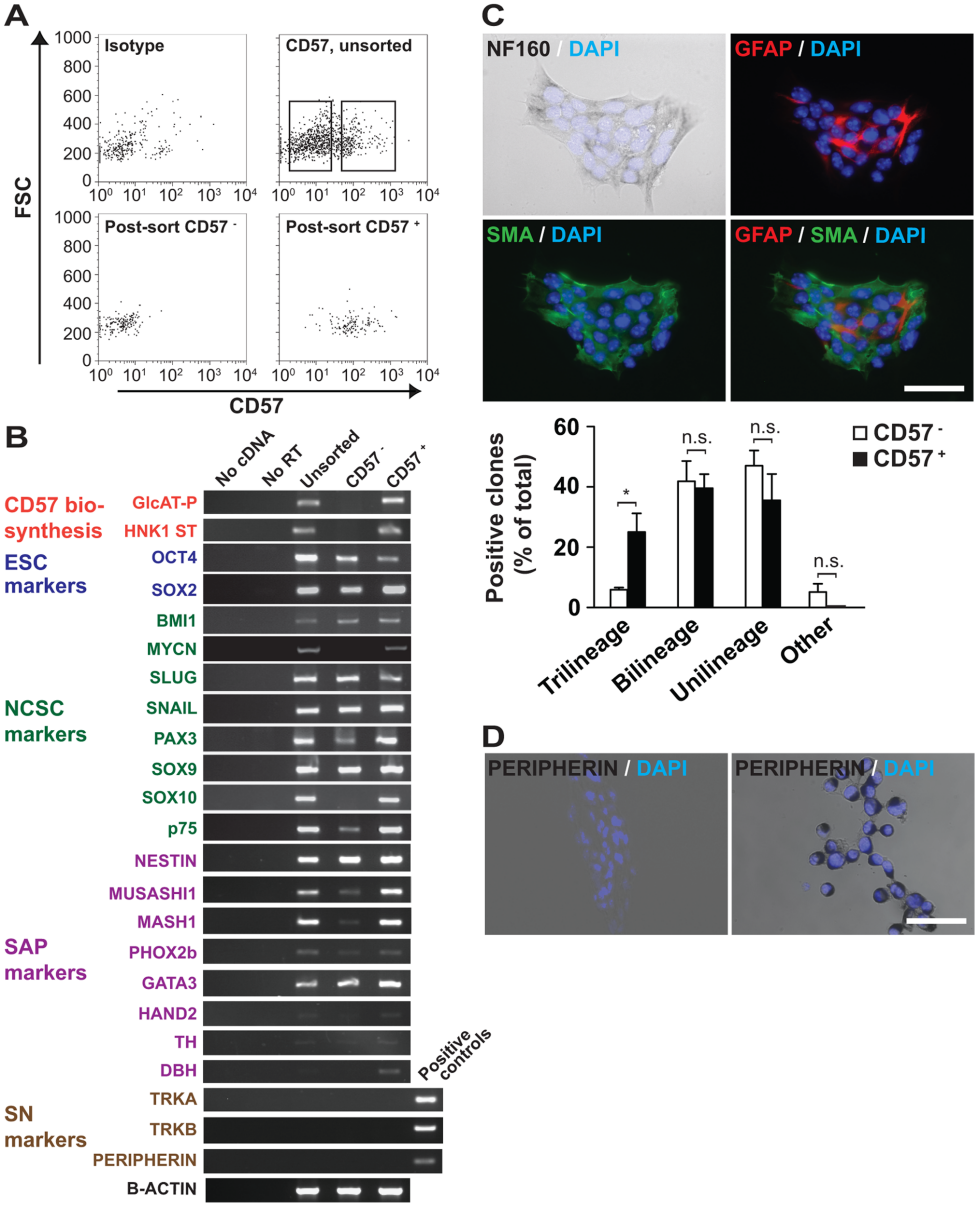
ESC-derived NCSC-like cells are enriched by sorting for expression of CD57. ESC-derived NPCs were cultured in medium containing chicken embryonic extract, retinoic acid, bFGF and IGF-1 under hypoxic conditions to generate low-enriched NCSC-like cells. (**A**) **A subset of low-enriched NCSC-like cells expresses CD57 and can be isolated by FACS.** Low-enriched NCSC-like cells were used. Left upper panel shows isotype control, right upper panel unsorted CD57-stained cells and lower panels post-sort plots of the CD57^−^ and CD57^+^ fractions. (**B**) **Stronger expression of some NCSC and SAP markers in CD57^+^ cells.** RNA isolated from unsorted, CD57^−^ and CD57^+^ cells was analyzed by RT-PCR to detect markers of CD57 biosynthesis, pluripotent cells, NCSCs, SAPs and sympathetic neurons. As positive control for TrkA and peripherin RT-PCR, cDNA from Neuro2a (a mouse NB) was used, while for TrkB mouse brain cDNA was employed. (**C**) **CD57^+^ cells have more trilineage NCSC potential as compared to the CD57** ^−^ **fraction.** CD57^−^sorted cells were cultured at clonal density in NCSC medium and resultant clones were simultaneously stained for NF160, GFAP, SMA and DAPI. A trilineage clone is shown. Scale bar equals 100 µm. The percentages of clones with cells of all three lineages, two lineages, one lineage or none of the lineage (“other”) were quantified. The means of three independent experiments (with a total of 183 clones) are depicted. Statistical analysis was performed using the t-test. *, p = 0.019. (**D**) **Clones from CD57^+^ cells do not contain cells expressing peripherin protein.** Clones (left panel) and neuro2a NB cells used as positive control (right panel) were stained for peripherin. Nuclei were counterstained with DAPI. Scale bar equals 100 µm.

As a first step to enrich for NCSC-like cells after NPC lineage selection, we cultured NPCs using conditions supportive for NCSCs. We reasoned that conditions employed for short-term maintenance of NCSCs from avian and rodent sympathetic ganglia and peripheral nerves might also enhance generation of ESC-derived NCSC-like cells. Thus, NPCs were seeded on poly-D-lysine/fibronectin-coated dishes and cultured for 7 d under hypoxia in medium containing chicken embryonic extract, bFGF, IGF-1 and retinoic acid (Fig. 1A). From this population with low-enriched NCSC-like cells, we sorted CD57 (HNK-1)-expressing cells to obtain a population of higher enriched NCSC-like cells (Fig. 1A). We chose CD57, as this carbohydrate epitope marks migrating NCSCs *in vivo* [2], [29]. The purity of the fractions was assessed by post-sort FACS analysis (Fig. 2A) and confirmed by expression analysis of glucuronyltransferase (GlcAT-P/B3GAT1) and sulfotransferase (HNK1ST), both of which specifically regulate biosynthesis of the CD57 carbohydrate (Fig. 2B).

RT-PCR analysis of genes associated with ESCs, NCSCs, SAPs and sympathetic neurons revealed that the unsorted putative low-enrichment NCSC-like population expressed genes characteristic for ESC, NCSCs and SAPs (Fig. 2B). Compared to CD57^−^ cells CD57^+^ cells expressed more of the NCSC markers PAX3, SOX10, p75, and of the SAP markers MUSASHI1 and MASH1. Genes crucial for SAPs such as PHOX2b, HAND2, TH and DBH were hardly expressed in CD57^+^ cells. None of the genes characterizing mature sympathetic neurons were expressed in the unsorted or sorted populations.

To determine lineage potential, CD57-sorted cells were cultured at clonal density in NCSC medium for 7 d. Clones were assessed by simultaneous staining for NF160 (neuronal lineage), GFAP (glial fibrillary acidic protein, glial lineage) and SMA (smooth muscle lineage). Significantly more clones from the CD57^+^ fraction compared to the CD57^−^ fraction were trilineage, i.e. originated from NCSC-like cells (Fig. 2C). Bilineage populations were positive for two markers and unilineage populations were positive for either NF160 or GFAP or SMA. The bilineage NF160/GFAP, NF160/SMA, SMA/GFAP or unilineage populations were present without prevalence of one population over the other. Immunocytology confirmed the absence of peripherin expression in clones from CD57^+^ cells (Fig. 2D).

Taken together, these data show that sorting of ESC-derived low-enriched NCSC-like cells for CD57 expression generates high-enriched NCSC-like cells but does not enrich for SAP-like cells.

### Sorting for GD2 Expression Enriches for ESC-derived SAP-like Cells

**Figure 3:**
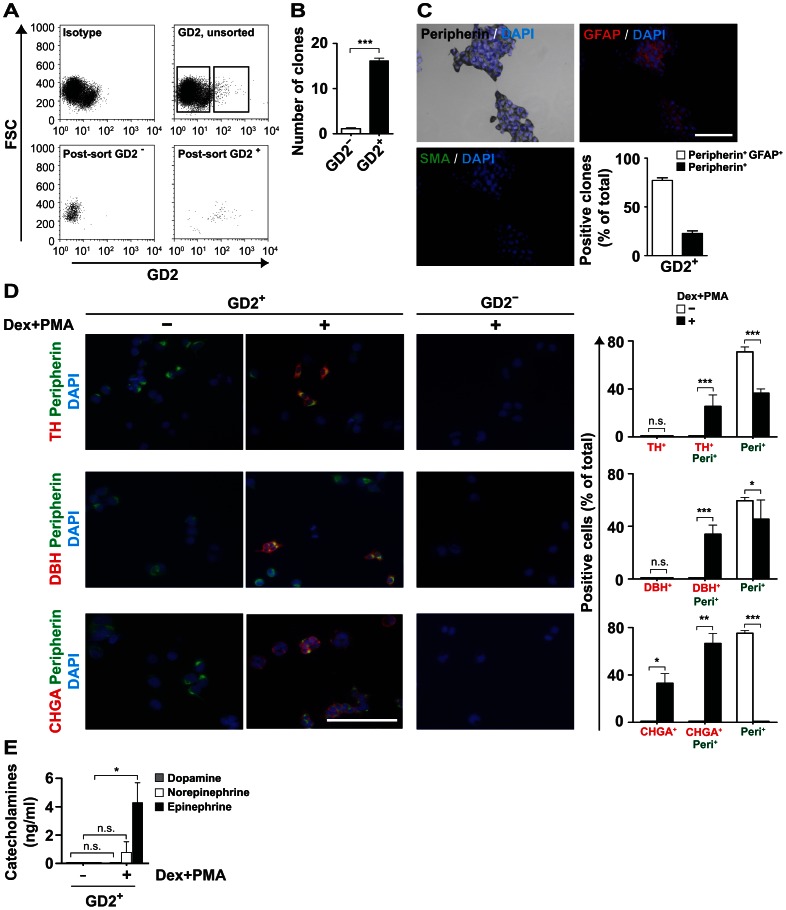
SAP-like cells can be enriched by sorting for GD2. (**A**) **A subset of the low-enrichment NCSC-like population expresses GD2.** ESC-derived low-enriched NCSC cultures were sorted into GD2^−^ and GD2^+^ fractions. Left upper panel shows the isotype control, right upper panel the GD2-stained population and the lower panels post-sort plots of the GD2^−^ and GD2^+^ fractions. (**B**) **GD2^+^ cells have a markedly higher clonogenicity than GD2** ^−^ **cells.** GD2-sorted cells were seeded at clonal density and clones were quantified after 7 d of culture in NCSC medium. The means of 3 independent experiments are depicted. Statistical analysis was performed using the t-test. ***, p<0.001. (**C**) **The large majority of GD2^+^ cells are bilineage SAP-like cells.** GD2-sorted cells were cultured at clonal density for 7 d in NCSC medium and resultant clones were simultaneously stained for peripherin, GFAP and SMA. A peripherin^+^GFAP^+^ clone is shown, scale bar equals 100 µm. Quantification of the clones is shown in the histogram, the means of three independent experiments (with a total of 219 clones) are depicted. (**D**) **Chromaffin cell-inducing conditions upregulate chromaffin markers in GD2^+^ cells.** GD2-sorted cells were differentiated for 6 d on poly-D-lysine/fibronectin-coated coverslips in NCSC medium containing dexamethasone and PMA for chromaffin cell induction. Immunocytochemistry images show GD2^+^ and GD2^−^ cells cultured with or without dexamethasone and PMA, simultaneously stained for TH and peripherin (upper panel), DBH and peripherin (middle panel), and chromogranin A and peripherin (lower panel). Nuclei were counterstained with DAPI. Scale bars equal 100 µm. Quantification of positive cells in the GD2^+^ fraction is shown in the histograms (2954 cells of two independent experiments were analyzed). Statistical analysis was performed using the t-test; n.s., not significant; *, p<0.05; **, p<0.01; ***, p<0.001. (**E**) **Chromaffin cell inducing conditions induce catecholamine production in GD2^+^ cells.** HPLC analysis of catecholamine content in lysates of GD2**^+^** cells differentiated for 6 d in NCSC medium supplemented with or without dexamethasone and PMA. The means of three independent experiments are shown. Statistical analysis was performed using the t-test; n.s., not significant; *, p<0.05.

GD2 is a ganglioside expressed on NB [30], [31], a tumor thought to originate from SAPs. We therefore investigated whether GD2 expression can be used to enrich for SAPs. GD2 was expressed on 4% of the cells of the low-enriched NCSC-like population (Fig. 3A). To determine lineage potential, GD2-sorted cells were cultured at clonal density for 7 d in NCSC medium. Peripheral neuron lineage was assessed by staining for peripherin, glial lineage by GFAP and mesenchymal lineage by SMA. Because of the near-absent clonogenicity of GD2^−^ cells (Fig. 3B) only clones from GD2^+^ cells could be assessed. Almost all GD2^+^ cells formed colonies with peripherin^+^ cells (Fig. 3C). The majority of clones also contained GFAP^+^ cells but no SMA^+^ cells. This shows that the majority of GD2^+^ cells have a bi-lineage potential towards peripheral neuronal and glial lineage while a minority of GD2^+^ cells are restricted to peripheral neuronal lineage.

To confirm the SAP-like status of GD2-sorted cells, GD2^+^ and GD2^−^ cells were subjected to differentiation *in vitro* with a combination of dexamethasone and PMA. This combinatorial treatment resulted in upregulation of chromaffin markers TH, DBH and chromogranin A (CHGA), thus demonstrating the chromaffin differentiation capacity of GD2^+^ cells (Fig. 3D). GD2^−^ cells were negative for neural and chromaffin markers and treatment with dexamethasone and PMA did not induce chromaffin marker expression (Fig. 3D). Importantly, HPLC analysis of sonicated cell lysates of GD2^+^ cells (differentiated with dexamethasone and PMA) showed upregulation in the levels of norepinephrine and epinephrine, thus indicating their chromaffin differentiation potential under chromaffin-inducing conditions (Fig. 3E). These data indicate, that sorting of ESC-derived low-enriched NCSC-like cells for GD2 expression highly enriches for SAP-like cells.

### Adrenal Glands of Postnatal Mice Contain a Population of Sphere-forming Cells Expressing Genes Characteristic of SAPs

**Figure 4:**
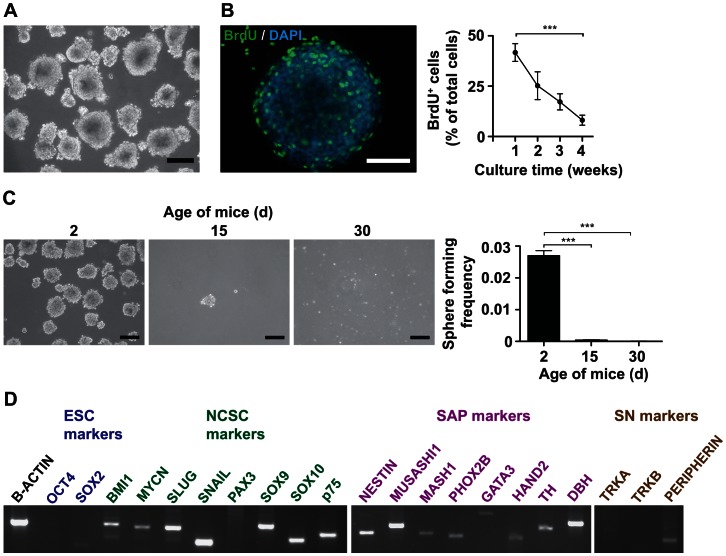
Postnatal mouse adrenal glands contain sphere-forming cells expressing markers of NCSCs and SAPs. Mouse adrenals were dissociated to single cells and cultured using differential plating in low-attachment plastic dishes with serum-free medium containing bFGF and EGF. (**A**) **Dissociated postnatal mouse adrenal glands form spheres in non-adherent serum-free conditions.** Phase contrast image of spheres derived from the adrenal glands of a 2 d old mouse growing in suspension culture. Scale bar equals 200 µm. (**B**) **Early spheres contain proliferating cells.** BrdU was added to the culture medium of spheres at different time of culture. 24 h later BrdU was detected by immunofluorescence microscopy. Micrograph of a sphere cultured for 7 d stained with anti-BrdU and DAPI is shown in the left panel, scale bar equals 50 µm. Quantification of BrdU-incorporating sphere cells is depicted in the right panel. The means of two independent experiments are shown (15 wells/time-point). Statistical analysis was performed using the t-test. ***, p<0.001. (**C**) **Frequency of sphere forming cells decreases with age of mice.** 1.2×10^5^ cells/ml derived from adrenal glands of mice of increasing age were seeded in non-adherent serum-free condition. Phase contrast images of spheres are shown, scale bar equals 200 µm. Sphere-forming frequency was calculated as frequency of spheres formed per number of cells seeded (histogram). The means of three independent experiments are shown. Statistical analysis was performed using the t-test. ***, p<0.001. (**D**) **Sphere cells express markers of SAPs and NCSCs.** RT-PCR analysis was performed on RNA isolated from adrenal-derived spheres.

Complementary to enriching SAP-like cells from ESCs, we set out to isolate and maintain SAP-like cells from the sympathetic PNS of postnatal mice. Initial attempts with superior cervical ganglions failed, as isolated ganglion cells stopped dividing and subsequently died within a few days after isolation (data not shown). Hence we turned our attention to the adrenal medulla. We cultured dissociated adrenal cells under serum-free and non-adhesive conditions as for neural stem cells. Under those conditions adrenal cells formed spheres after 3–6 d (Fig. 4A). Spheres contained proliferating, i.e. BrdU^+^ cells, the percentage of proliferating cells in the spheres significantly decreased over four weeks in continuous culture (Fig. 4B).

Adrenal-derived spheres could only be generated in higher numbers from 2–3 d old mice; adrenals from older mice yielded significantly less or no spheres (Fig. 4C). The frequency of sphere-forming adrenal cells was low (Fig. 4C). Spheres could be passaged for up to two passages (data not shown), suggesting limited self-renewal of sphere cells.

Next we investigated the expression pattern of genes characteristic for pluripotent cells, NCSCs, SAPs and sympathetic neurons (Fig. 4D). While the pluripotency-associated genes OCT4 and SOX2 were not expressed, genes associated with NCSCs were expressed. Importantly, all genes characteristic for SAPs were expressed, including TH and DBH.

Taken together, these data show that spheres from early postnatal adrenal glands of mice contain rare proliferating cells with limited self-renewal that express genes associated with SAPs and NCSCs.

### Adrenal Spheres Contain Many SAP-like Cells and Some Sympathetic Neurons

As GD2 expression led to identification of SAP-like cells in differentiated ESCs, we asked if GD2 could be used as a marker to identify SAP-like cells from adrenal-derived spheres. Adrenal-derived spheres as well as postnatal adrenal gland cells did not express GD2 (Fig. S1), in line with the absence of GD2 expression in normal human tissues including the fetal adrenal gland [30]. To analyze if culture conditions used for ESC-derived sorted cells could impart SAP-like properties to adrenal-derived spheres, we attempted to culture the spheres adherently in NCSC media. Unlike ESC-derived cells, the spheres did not tolerate NCSC medium (Fig. S2).

As the spheres were derived from entire dissociated adrenal gland (composed of cortex and medulla), analysis of adrenocortical markers (SF-1, CYP11A1, CYP11B2) and endothelial marker CD31 was carried out to rule out the presence of adrenocortical and endothelial cells in the spheres. Indeed, the spheres did not express these adrenocortical and endothelial markers (Fig. S3).

**Figure 5:**
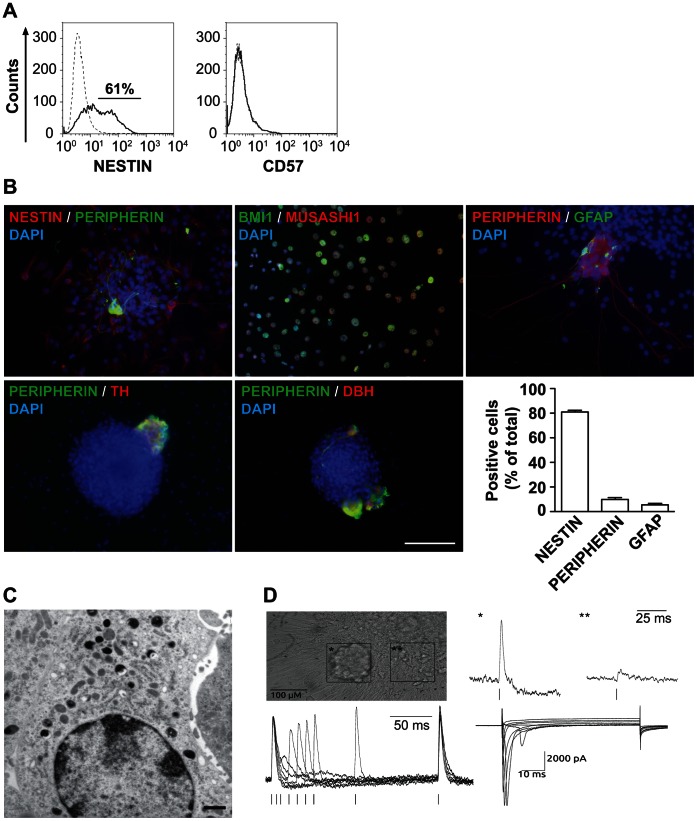
Adrenal spheres contain many SAP-like cells and few sympathetic neurons and glial cells. (**A**) **A majority of sphere cells express nestin, while none express CD57.** Flow cytometric analysis of dissociated adrenal-derived sphere cells stained for nestin (left panel) and CD57 (right panel). Dashed lines define isotype controls and solid lines specific antigen expression. (**B**) **The majority of adrenal-derived sphere cells expresses nestin, BMI1 and MUSASHI1 while few cells express peripherin or GFAP.** Immunofluorescence microscopy of spheres allowed to attach overnight on poly-D-lysine/fibronectin-coated glass coverslips in 1% FCS-containing sphere medium, analyzed for co-expression of nestin, peripherin, BMI1, MUSASHI1, GFAP, TH, DBH. Nuclei were counterstained with DAPI. Scale bar equals 100 µm. Quantification of positive cells is shown in the histogram. (**C**) **Adrenal-derived spheres contain a minority of cells with dense core vesicles.** Representative electron microscopic image of a cell from an adrenal-derived sphere. Scale bar equals 1 µm. (**D**) **Adrenal-derived spheres harbor many immature appearing cells lacking functional voltage-gated channels and few mature appearing neurons exhibiting electrical activity.** Microphotograph showing a neuronal-like cell cluster (*) and an area outside of the cluster (**). Fluorescence change (ΔF) in di-8-ANEPPS stained preparations corresponding to compound action potentials (CAP) from the cluster (*) and lack of CAP in the area outside of it (**), upper right panel. Representative traces of double pulse stimulation, whereby the second CAP was elicited at Δt of 100, 50, 40, 30, 20, 10, and 5 ms, lower left panel. Representative voltage-activated whole-cell inward currents recorded from a cell depolarized in 5 mV steps from –70 to +35 mV for 60 ms, from a holding potential of −120 mV, lower right panel.

We set out to delineate the cellular composition of the spheres. FACS analysis revealed that the majority of the sphere cells consisted of nestin-positive neural progenitors, while cells expressing CD57 were absent (Fig. 5A). Immunofluorescence microscopy of spheres allowed to attach confirmed the preponderance of cells expressing nestin, BMI1 and MUSASHI1, markers associated with SAPs (Fig. 5B). Sphere cells lacked both basal and dexamethasone-induced expression of chromogranin A and phenylethanolamine N-methyltransferase, markers of chromaffin cells (data not shown). Few peripherin^+^, TH- and DBH-expressing sympathetic neurons were present, and GFAP^+^ cells were rare. The presence of a large number of nestin^+^ cells and the rarity of cells expressing TH or DBH is consistent with most nestin^+^ cells not expressing TH or DBH. Along this line, electron microscopy revealed that only few sphere cells (9%) contained chromaffin granules, i.e. catecholamine storing dense core vesicles found in mature chromaffin cells (Fig. 5C). In adrenal-derived spheres, SCN7A encoding NaV2.1 channel was expressed, along with SCN2A, SCN3A, and SCN9A that encode for other sodium channels (Fig. S4). This suggested that some of the adrenal-derived sphere cells might be able to generate action potentials. Indeed, the few clusters containing neurons with prominent neurites (1–2 cluster per sphere, 5%) that formed from attached spheres showed responses to electrical stimulation (Fig. 5D). Apart from the neuronal-clusters, there were many (95%) immature-appearing cells, i.e. small and flat cells without neurites that were electrophysiologically inactive (Fig. 5D). Double pulse experiments determining the refractory period proved the signals to be compound action potentials (Fig. 5D). In accordance, whole-cell patch clamp measurements showed abundant voltage-gated sodium current which are necessary to initiate the action potential (Fig. 5D). Peak I–V relationship of fast inward currents was typical for voltage-gated Na^+^-channels (data not shown). Thus, electrophysiological analysis showed functional maturation in the few neuronal cell clusters, as they could fire action potentials and could generate typical Na^+^-mediated inward currents.

Taken together, these data show that adrenal-derived spheres contained a large proportion of proliferating cells that express SAP-associated genes. In contrast, only few cells possessed markers, enzymes, catecholamine storage vesicles and electric excitability typical of mature chromaffin cells and sympathetic neurons.

### Neural and Chromaffin Induction of Adrenal-derived Spheres Confirms their SAP-like Differentiation Capacity

**Figure 6:**
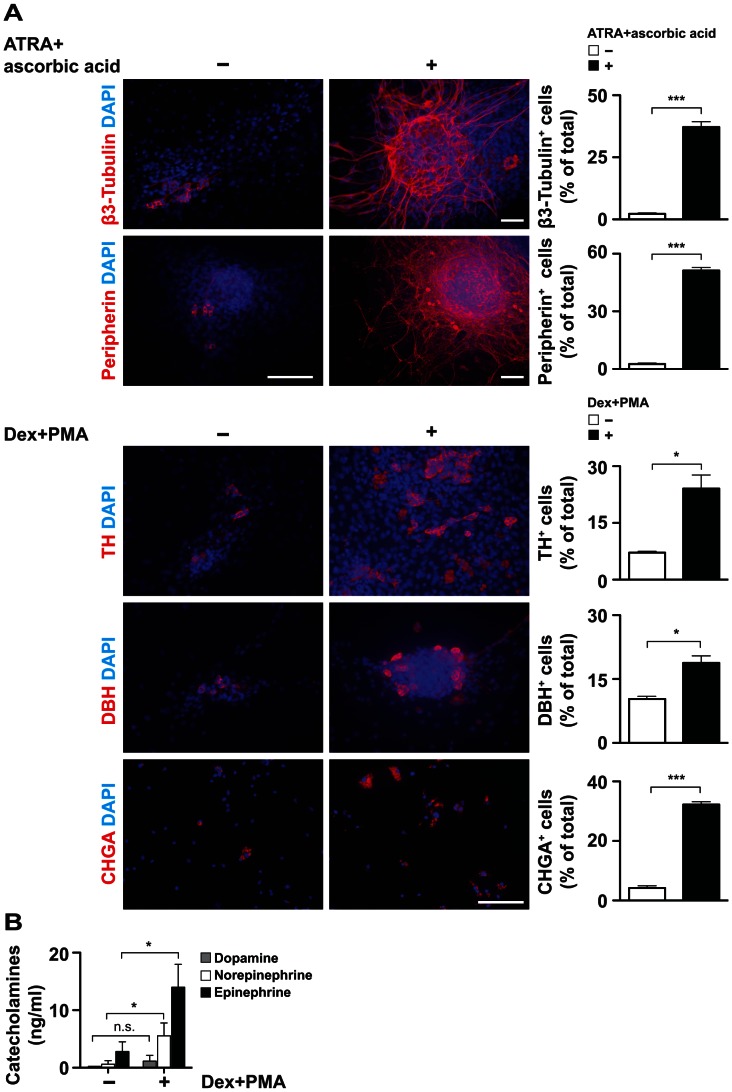
Adrenal-derived sphere cells can be induced *in vitro* towards neuronal and chromaffin lineage. (**A**) **Neuronal and chromaffin differentiation of spheres.** 7 d old spheres adhering to poly-D-lysine/fibronectin-coated coverslips were differentiated for 6 d in differentiation medium containing ATRA and ascorbic acid (for neuronal differentiation) or dexamethasone and PMA (for chromaffin cell induction). Immunocytochemistry images show spheres cultured with or without specific factors as indicated and stained for β3-Tubulin, peripherin, TH, DBH and CHGA. Nuclei were counterstained with DAPI. Scale bars equal 100 µm. Quantification of positive cells is shown in histograms (1994 cells of two independent experiments were analyzed). Statistical analysis was performed using the t-test. *, p<0.05; **, p<0.01; ***, p<0.001. (**B**) **Chromaffin cell inducing conditions upregulate catecholamines in spheres.** HPLC analysis of catecholamine content in lysates of spheres differentiated in adherence for 6 d in differentiation medium supplemented with or without dexamethasone and PMA. The means of three independent experiments are shown. Statistical analysis was performed using the t-test; n.s., not significant; *, p<0.05.

*In vitro* differentiation capacity of adrenal-derived spheres was analyzed by differentiating them adherently for 6 d with a combination of ATRA and ascorbic acid for neural differentiation, and with a combination of dexamethasone and PMA for chromaffin differentiation. As evident from Fig. 6A, differentiation with a combination of ATRA and ascorbic acid induced neural differentiation and formation of complex neurite-network post-differentiation as determined by the expression of β3-Tubulin and peripherin. Differentiation with a combination of dexamethasone and PMA resulted in upregulation of chromaffin markers TH, DBH and CHGA (Fig. 6A). In line, HPLC analysis of sonicated cell lysates of adrenal-derived spheres differentiated with dexamethasone and PMA showed upregulation of catecholamines (Fig. 6B). Taken together, the differentiation capacity of spheres towards neuronal and chromaffin cells shows that the majority of adrenal sphere cells have characteristics of SAP-like cells.

**Figure 7:**
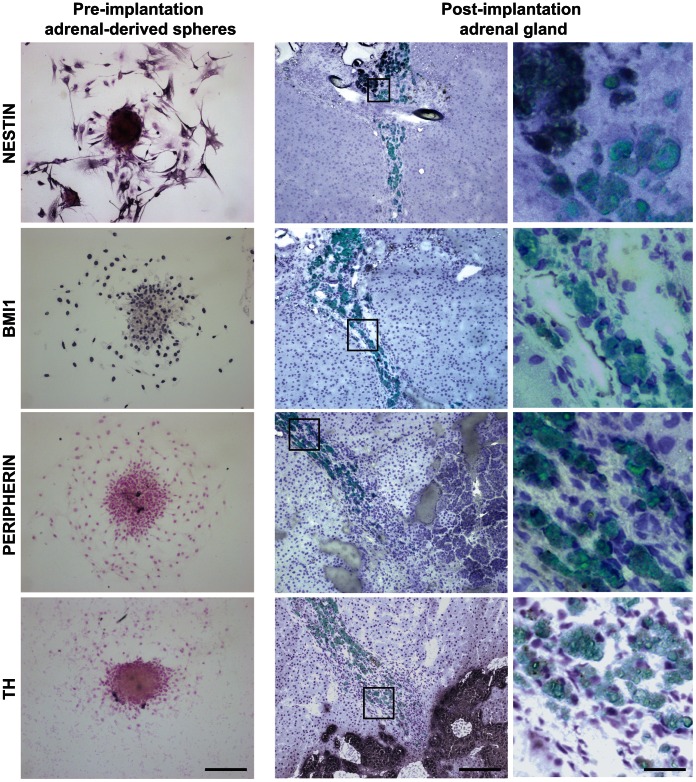
Adrenal-derived sphere cells integrate *in situ* where they downregulate expression of progenitor markers but do not differentiate to chromaffin cells. Pre-implantation adrenal-derived spheres attached to glass coverslips (left column) and 3 week post-implantation rat adrenal gland prior implanted with CFSE-labeled sphere cells (middle and right columns) were stained for nestin, BMI1, peripherin and TH. Stainings were visualized by immunohistochemistry. In the middle and right columns, fluorescent images (CFSE, green) are overlaid on immunohistochemistry images. Images in the right column are magnifications of images shown in the middle column. Scale bars are 200 µm (left and middle columns) and 50 µm (right column).

To assess whether adrenal-derived sphere cells survive *in situ*, we transplanted CFSE-labeled sphere cells into adrenal glands of immunodeficient rats. Analysis after three weeks of transplantation showed that sphere cells had survived and integrated into the cortical-medullary border of the adrenals (Fig. 7). While at that time the progenitor marker nestin was partially downregulated and BMI1 became undetectable in the transplanted cells, peripherin and TH were not induced (Fig. 7). Taken together, these data show the capacity of adrenal-derived sphere cells to integrate into the adrenal gland without differentiating towards chromaffin cells.

## Discussion

SAPs play an important role in development of the sympathetic PNS and are putative cells of origin of neuroblastoma. Efforts have been made to isolate these cells from PNS tissues of rodents such as sciatic nerve, gut or sympathetic ganglia [32]–[36], and from bone marrow, carotid body, cornea, dental tissue, dorsal root ganglion, heart, palatum and skin of adult rodents [6]. As isolation of NCSCs and SAPs is hampered by the small number and limited life span of the cells that can be procured, murine neural tube explant cells have been immortalized by oncogenes [37], [38]. To obtain NCSCs and SAPs without the potentially confounding effects of immortalization, NCSC-like and SAP-like cells have been differentiated from murine embryonic stem cells [39]–[44], human ESC [45]–[48] and human iPS [49]. Fetal noradrenaline-secreting chromaffin cells isolated from human fetuses could be propagated as neurospheres [50], [51]. However, access to this source is limited for logistical and ethical reasons.

As SAPs remain elusive we aimed to advance their differentiation them from murine ESCs and to isolate them from adrenal glands of postnatal mice. In this paper we show that ESC-derived SAP-like cells can be enriched by sorting for expression of GD2. Using a complimentary approach we enriched proliferating SAP-like cells from postnatal adrenal glands of mice.

Subjecting mouse ESCs to culture conditions successfully used to select NPCs, cells were generated that expressed genes associated with NCSCs and SAPs. This suggested that within this heterogeneous cell population not only NPCs but also NCSC-like and SAP-like cells might be present. We thus adapted culture conditions to support NCSCs and sorted the resulting low-enriched NCSC-like cell population for expression of CD57. This led to higher-enriched NCSC-like cells, but not SAP-like cells, in line with CD57 defining migrating NCSCs [29].

To enrich for ESC-derived SAP-like cells we sorted low-enriched NCSC-like cells for GD2-expressing cells. While the role of GD2 in the development of the sympathetic PNS is unknown, GD2 is expressed in virtually all NB, mainly in neuroblastic cells [30], [31]. We reasoned that GD2 might thus be also expressed on the putative cell of origin of NB, the SAP. Indeed, clonal lineage determination of the rare GD2^+^ cells showed them to be highly enriched for progenitors restricted to SAP-like lineage. Challenging GD2^+^ cells *in vitro* under chromaffin-inducing conditions led to their differentiation towards chromaffin cells with the upregulation of catecholamine content. It cannot be excluded that some transdifferentiation events might have occurred during *in vitro* differentiation.

It has to be noted that our conclusions about lineage are based on single markers and on forced differentiation *in vitro* using medium conditions not completely defined. This is why we use the terms NCSC-like and SAP-like cells. Furthermore, as the cell populations are heterogeneous, additional steps will be required to generate pure ESC-derived NCSC-like cells and SAP-lineage competent progenitors. These caveats notwithstanding, the ability to generate SAP lineage-competent progenitors with moderate heterogeneity from murine ESCs opens new avenues to probe the functions of these progenitors and the consequences of their malignant transformation.

Generation of SAP-like cells from ESCs is hampered by cellular heterogeneity and these cells may differ from *in situ* SAPs. We therefore aimed to isolate SAP-like cells from the adrenal medulla of postnatal mice. We succeeded in isolating and expanding proliferating SAP-like cells with limited self-renewal that lacked the enzymes, catecholamine storage vesicles and electric excitability of mature chromaffin cells. To this end we used similar conditions recently described for generating chromaffin progenitors from the adrenals of adult cattle [25], [52], [53]. There, chromaffin progenitors were enriched by differential plating and culturing as ‘chromospheres’ under non-adherent conditions. Using dexamethasone, or ATRA with ascorbic acid, chromosphere-derived cells could be differentiated to mature chromaffin cells and dopaminergic neurons, respectively. Given the small size of mouse adrenal gland compared to cattle adrenals, we used whole adrenals rather than dissected medullae. Differential plating of dissociated adrenals and non-adherent growth as spheres in serum-free medium ensured that no adrenocortical or endothelial cells were present in the spheres. Mouse adrenal-derived spheres expressed progenitor markers similar to bovine chromospheres. Like chromospheres, mouse adrenal-derived spheres, that were predominantly nestin-positive, could be efficiently differentiated to chromaffin cells and sympathetic neurons, suggesting the existence of a common bipotential progenitor. Unlike bovine chromaffin progenitors, murine adrenal-derived SAPs were not found in adult animals. This might be explained by species differences such as very low number of sphere-forming progenitors found in the mouse adrenal, specificities of the mouse strain used, and methodological differences in procuring the adrenal cells.

The frequency of adrenal cells able to generate spheres containing SAP-like cells was very low, notwithstanding that many of the cells put into the sphere forming assays were cortical cells destined to die under the conditions of the assay. In addition, the ability of cells to generate spheres was limited to adrenals from mice just several days old. Considering the ability to generate spheres as a characteristic of progenitors, this suggests that a minority of adrenal medullary cells in mice are progenitor cells and that they mature rapidly after birth. The latter is in line with the rapid postnatal decrease of nestin-positive proliferating cells in sympathetic superior cervical ganglia of mice [54]. SAP-like cells in spheres showed limited self-renewal and lifespan, consistent with their progenitor status.

*In vitro* differentiation of spheres towards neural and chromaffin cells showed their SAP-like differentiation capacity. *In vivo*, after orthotopic transplantation into the rat adrenal gland, the bulk of transplanted sphere cells exhibited normal morphology and retained CFSE dye, suggestive of an intact cytoskeleton as well as membrane integrity, therefore confirming post-engraftment survival and integration *in situ*. Upon transplantation, the progenitor marker nestin was partially and BMI1 was completely downregulated. There was no concomitant up-regulation of the neuronal or catecholaminergic markers, peripherin and tyrosine hydroxylase, respectively. This absence of differentiation *in situ* might have been caused by disturbance of the adrenal niche by the microsurgical procedure or by ultrastructural incompatibility between mouse and rat tissue. Alternatively, the *in vivo* differentiation capacity of adrenal sphere-derived SAP-like cells might be limited.

Taken together, in both of our *in vitro* strategies using murine ESCs and adrenal-derived spheres we were able to derive SAP-like cells.

### Conclusions

We have described improved complementary approaches that generate cell populations enriched for SAP-like cells from murine ESC and from mouse adrenals. This facilitates experimental access to SAPs, cells important for the normal and abnormal development of the sympathetic PNS, in mice, the preeminent animal model for biomedical research.

## Supporting Information

Figure S1 **GD2 is not expressed in adrenal-derived sphere cells.** Flow cytometric analysis of GD2 expression in adrenal gland of 2 d old mice and 7 d old adrenal-derived sphere cells. Mouse neuroblastoma cells from TH-MYCN mice were used as positive staining control.
(TIF)

Figure S2 **Adrenal-derived spheres do not tolerate NCSC medium.** Spheres cultured for 4 d in basal media (upper panel) and NCSC medium (lower panel) are shown. Scale bar equals 100 µm.
(TIF)

Figure S3 **Adrenal-derived spheres do not contain adreno-cortical and endothelial cells.** Immunohistochemical analysis of freshly dissociated 2 d old mouse adrenal glands (cytospin, left panel) showing expression of cortical markers SF-1, CYP11A1, CYP11B2 and endothelial marker CD31. Adrenal-derived spheres stained for the same markers are shown in right panel (cryocuts). HRP-conjugated secondary antibodies were developed by diaminobenzidine, nuclei were counterstained with hematoxylin. Scale bar equals 100 µm.
(TIF)

Figure S4 **Adrenal-derived spheres express genes encoding voltage-gated sodium channels.** RT-PCR analysis of voltage-gated sodium channels in adrenal-derived spheres. RNA from mouse mixed tissue lysate (pancreas, heart, muscle, brain, liver, kidney) was used as a positive control.
(TIF)

Table S1 **Primary and secondary antibodies.**
(DOC)

Table S2 **Primer sequences.**
(DOC)
