# Supplementary figures and images for: Generation of Murine Sympathoadrenergic Progenitor-Like Cells from Embryonic Stem Cells and Postnatal Adrenal Glands
Source: PLoS One. 2013 May 10;8(5):e64454. doi: 10.1371/journal.pone.0064454 (PMC3651195; doi:10.1371/journal.pone.0064454)

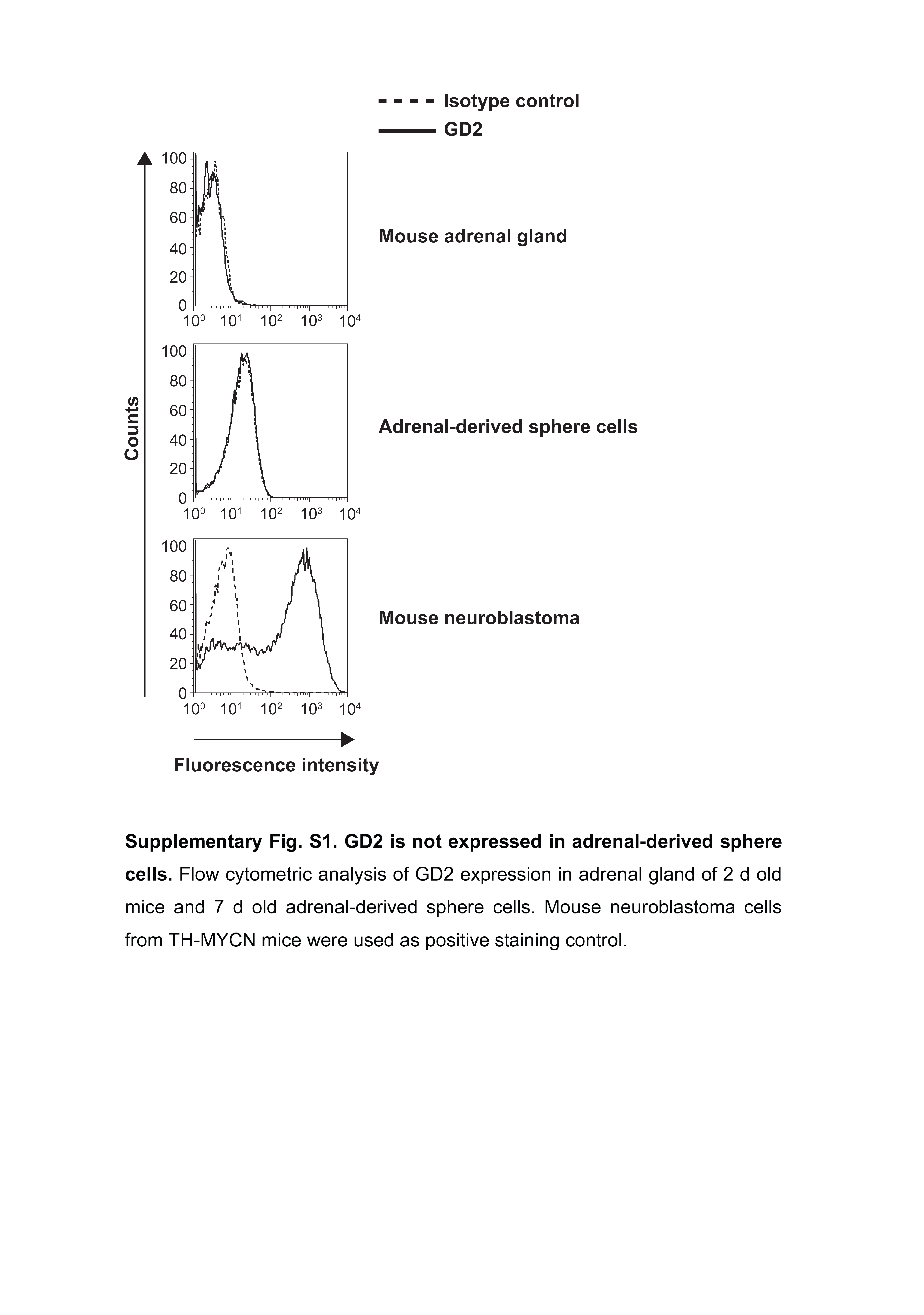

Supplement: Figure S1 — GD2 is not expressed in adrenal-derived sphere cells. Flow cytometric analysis of GD2 expression in adrenal gland of 2 d old mice and 7 d old adrenal-derived sphere cells. Mouse neuroblastoma cells from TH-MYCN mice were used as positive staining control. (TIF) [file pone.0064454.s001.tif]

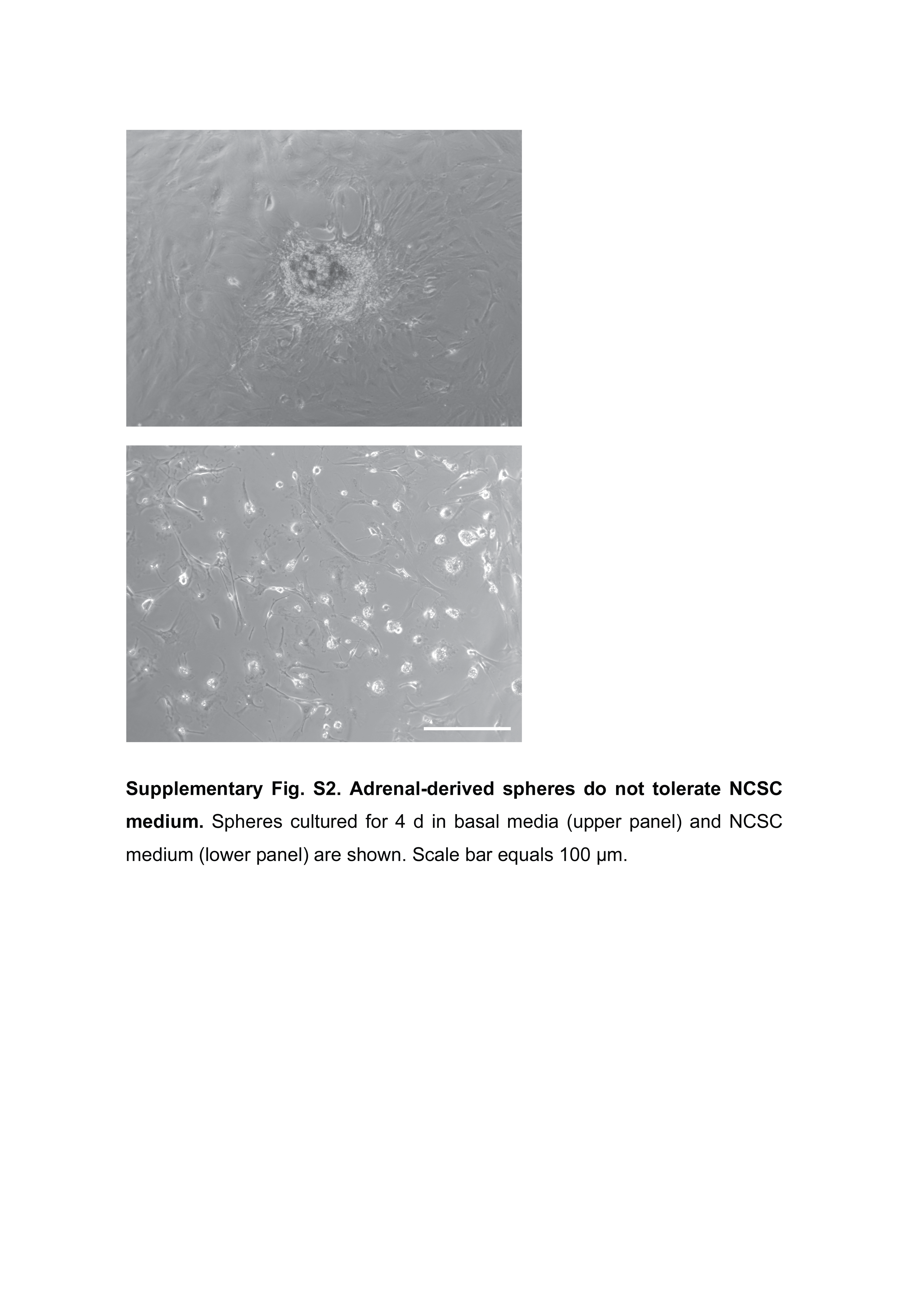

Supplement: Figure S2 — Adrenal-derived spheres do not tolerate NCSC medium. Spheres cultured for 4 d in basal media (upper panel) and NCSC medium (lower panel) are shown. Scale bar equals 100 µm. (TIF) [file pone.0064454.s002.tif]

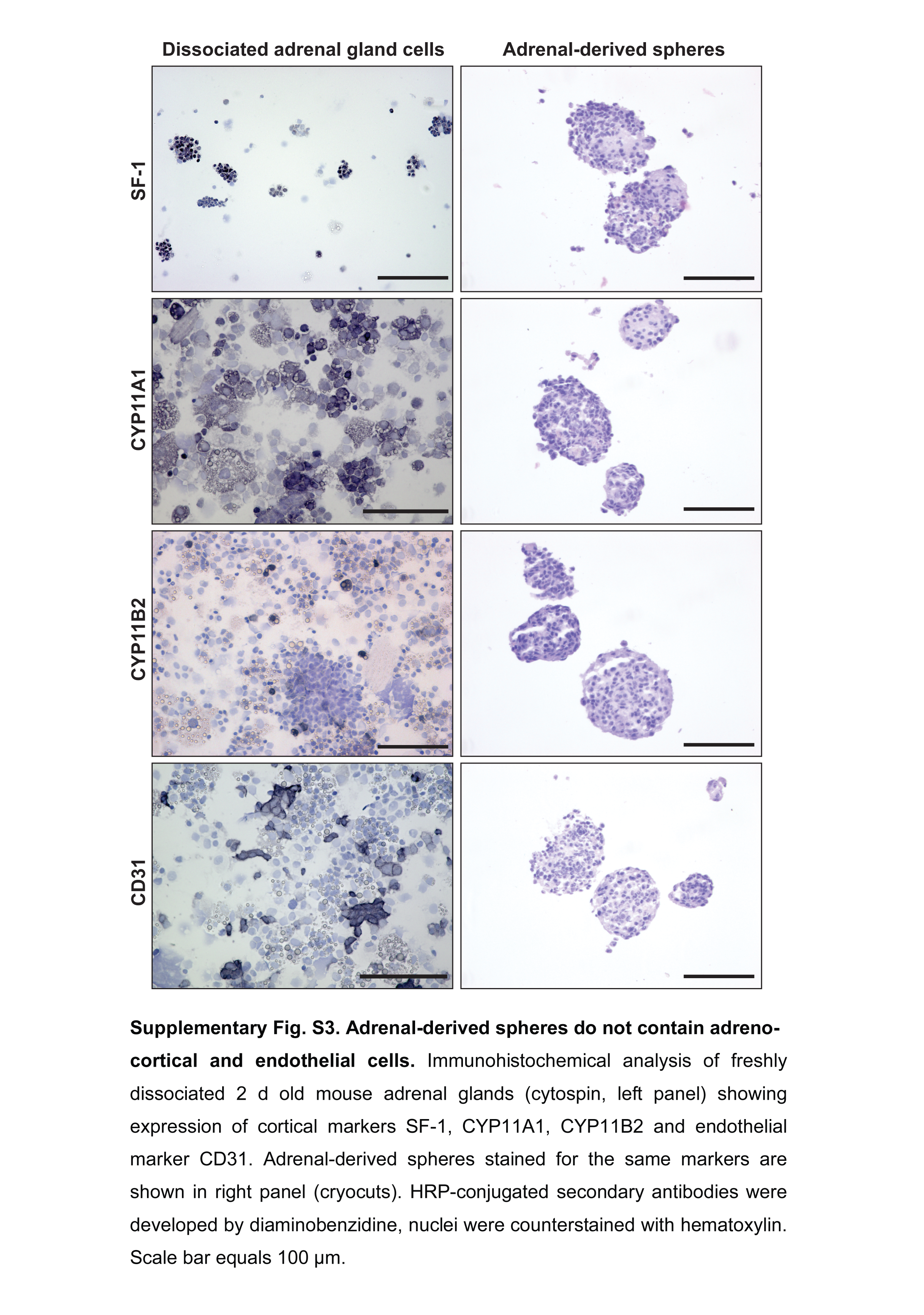

Supplement: Figure S3 — Adrenal-derived spheres do not contain adreno-cortical and endothelial cells. Immunohistochemical analysis of freshly dissociated 2 d old mouse adrenal glands (cytospin, left panel) showing expression of cortical markers SF-1, CYP11A1, CYP11B2 and endothelial marker CD31. Adrenal-derived spheres stained for the same markers are shown in right panel (cryocuts). HRP-conjugated secondary antibodies were developed by diaminobenzidine, nuclei were counterstained with hematoxylin. Scale bar equals 100 µm. (TIF) [file pone.0064454.s003.tif]

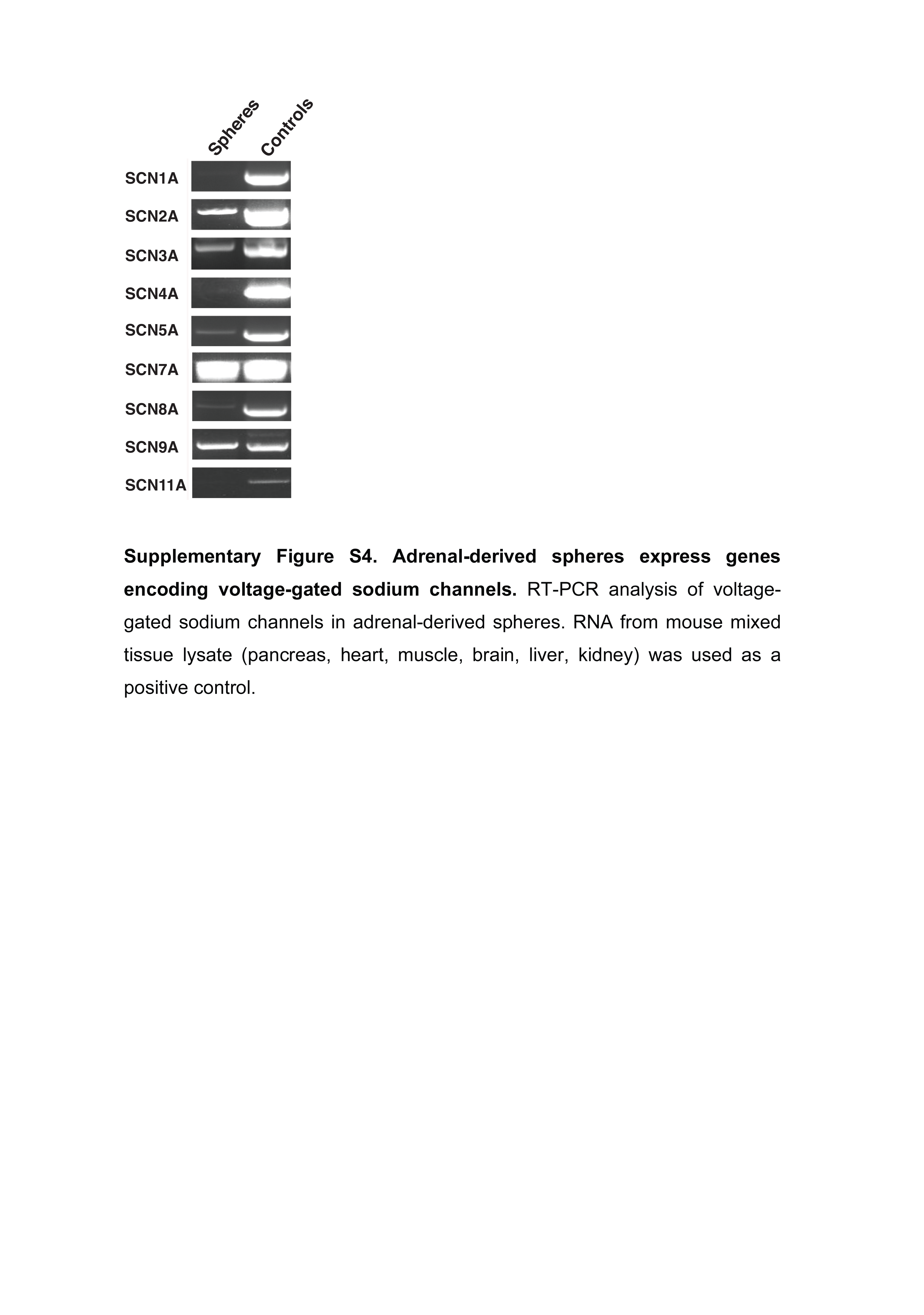

Supplement: Figure S4 — Adrenal-derived spheres express genes encoding voltage-gated sodium channels. RT-PCR analysis of voltage-gated sodium channels in adrenal-derived spheres. RNA from mouse mixed tissue lysate (pancreas, heart, muscle, brain, liver, kidney) was used as a positive control. (TIF) [file pone.0064454.s004.tif]
