# Supplementary material for: Generation of Murine Sympathoadrenergic Progenitor-Like Cells from Embryonic Stem Cells and Postnatal Adrenal Glands
Source: PLoS One. 2013 May 10;8(5):e64454. doi: 10.1371/journal.pone.0064454 (PMC3651195; doi:10.1371/journal.pone.0064454)
Supplement: Table S1 — Primary and secondary antibodies. (DOC) [file pone.0064454.s005.doc]

**Supplementary Table 1.** Primary and secondary antibodies.

| **Primary antibodies** | **Host / Isotype** | **Company** | **Dilution** |
| --- | --- | --- | --- |
| Anti-Peripherin | Rabbit polyclonal | Millipore | 1:1000 |
| Anti-Peripherin (clone 8G2) | Mouse IgG_1_ | Sigma-Aldrich | 1:1000 |
| Anti-NF160 | Rabbit polyclonal | Abcam | 1:500 |
| Anti-β3-Tubulin | Rabbit polyclonal | Covance, Inc.  Munich | 1:1000 |
| Anti-Nestin (clone rat-401) | Mouse IgG_1_ | Developmental Hybridoma Bank, Iowa, USA | 1:200 |
| Anti-GFAP (clone GA5) | Mouse IgG_1_ | Sigma-Aldrich | 1:500 |
| Anti-SMA (clone 1A4) | Mouse IgG_2a_ | Sigma-Aldrich | 1:400 |
| Anti-TH | Rabbit polyclonal | Pel-Freez, Rogers, AR, USA | 1:500 |
| Anti-DBH | Rabbit polyclonal | ImmunoStar, Inc. Hudson, WI, USA | 1:200 |
| Anti-BMI1 (clone F6) | Mouse IgG_1_ | Millipore | 1:400 |
| Anti-MUSASHI1 | Rabbit polyclonal | Millipore | 1:500 |
| Anti-Chromogranin A | Rabbit  polyclonal | Origene Technologies, MD | 1:250 |
| Anti-SF1 | Rabbit polyclonal | Sigma-Aldrich | 1:50 |
| Anti-CYP11A1 | Rabbit polyclonal | Santa Cruz Biotechnology, CA | 1:25 |
| Anti-CYP11B2 | Mouse IgG_1_ | Millipore | 1:50 |
| Anti-CD31 | Rat IgG_2a_ | Dianova, Hamburg | 1:20 |
| Anti-CD57 | Mouse IgM | Abcam | 0.1 µg / 1x10^6^ cells |
| Anti-GD2 (clone 14.G2a) | Mouse IgG_2a_ | BD Biosciences | 0.5 µg / 1x10^6^ cells |
| **Secondary antibodies** |  |  |  |
| Anti-Rabbit IgG_1_ HRP | Goat | Life Technologies, Germany | 1:500 |
| Anti-Rabbit IgG Alexa 488 | Goat | Life Technologies | 1:400 |
| Anti-Rabbit IgG Alexa 594 | Goat | Life Technologies | 1:400 |
| Anti-Mouse IgG Alexa 488 | Goat | Life Technologies | 1:400 |
| Anti-Mouse IgG Alexa 594 | Goat | Life Technologies | 1:400 |
| Anti-Mouse IgG_1_ HRP | Goat | Santa Cruz | 1:500 |
| Anti-Mouse IgG_2a_ FITC | Goat | Southern Biotech,  Alabama, USA | 1:400 |
| EnVision^TM+^ Dual Link polymer-HRP | Goat | Dako, Glostrup, Denmark | 30 µl per section |
