# Supplementary material for: Generation of Murine Sympathoadrenergic Progenitor-Like Cells from Embryonic Stem Cells and Postnatal Adrenal Glands
Source: PLoS One. 2013 May 10;8(5):e64454. doi: 10.1371/journal.pone.0064454 (PMC3651195; doi:10.1371/journal.pone.0064454)
Supplement: Table S2 — Primer sequences. (DOC) [file pone.0064454.s006.doc]

**Supplementary Table 2.** Primer sequences.

| **Gene / Accession number** | **Primer sequences**  **(F = forward, R = reverse)** | **Amplicon / annealing temperature** |
| --- | --- | --- |
| BMI1  NM_007552 | **F** GAT TGA CGT CAT GTA TGA AGA GG  **R** A ACC AGA TGA AGT TGC TGA TGA CC | 410 bp, 64°C |
| MycN  NM_008709 | **F** GAG AGG ATA CCT TGA GCG ACT C  **R** CTC GCT GTC CTC CGA GTC TGA G | 375 bp, 64°C |
| Sox2  NM_011443 | **F** CAC AAC TCG GAG ATC AGC AA  **R** CTC CGG GAA GCG TGT ACT TA | 190 bp, 57°C |
| Oct4  NM_013633 | **F** AGC TGC TGA AGC AGA AGA GG  **R** TGA TTG GCG ATG TGA GTG AT | 356 bp, 57°C |
| DβH  NM_138942 | **F** ACC CGG GGG ACG TAC TCA TCA C  **R** CAA AGG CTG CAG GTT CCA CTC AC | 377 bp, 62°C |
| TH  NM_009377 | **F** CTG TGG AGT TTG GGC TGT GTA A  **R** CGC CGG ATG GTG TGA GGA CTG T | 313 bp, 62°C |
| Hand2  NM_010402 | **F** AGC GGC GCA GGA CTC AGA GCA TCA  **R** CTC TTC TCC TCT TTC ACG TCG GTC | 220 bp, 60°C |
| Phox2b  Y14493 | **F** TCA ACC CCA CTC CTA CCC CTT TCC  **R** TCA AGT TGG TTG TGG TGC CCC CGT | 243 bp, 60°C |
| GATA3  NM_008091 | **F** CAG CCC ACC ACC CCA TTA CCA CCT  **R** GTC CTC CAG CGC GTC ATG CAC CTT | 515 bp, 60°C |
| NESTIN  NM_016701 | **F** CAC CCA TCC CGA TTT TGA CAG  **R** TCC TCC AGC TTG CTC CCA CTT TG | 278 bp, 64°C |
| Musashi1  NM_008629 | **F** ACT CAG TTG GCA GAC CAC GCA G  **R** TGT CGA ACA TCA GCA TGG CAT C | 352 bp, 63°C |
| Sox9  NM_011448 | **F** CTG CAA GCC GAC TCC CCA CAT TCC  **R** GCG CCT GCT GCT TCG ACA TCC AC | 409 bp, 63°C |
| Sox10 XM_128139 | **F** GGG GAG ATC AGC CAC GAG GTA ATG  **R** TC CCT GGG GGC CTG TGG TCT CTG | 259 bp, 63°C |
| MASH1 NM_008553 | **F** GAA CCT AAG CCC GAA TCA C  **R** GAA AGG CTG TCC GAG AAC T | 271 bp, 60°C |
| Slug  U97059 | **F** ATG CCG CGC TCC TTC CTG  **R** GAT GGC ATG GGG GTC TGA | 372 bp, 60°C |
| Snail  X67253 | **F** GCC TGG GCG CTC TGA AG  **R** AGG CCT GGC ACT GGT ATC TCT | 228 bp, 60°C |
| Pax3 NM_008781 | **F** GAG GAA GCA GCG CAG GAG CAG AAC  **R** GGC ATG GCG GTG GGA GGG AAT C | 252 bp, 59°C |
| p75  NM_033217 | **F** TGG CCG ATG GAT CAC AAG GTC TAC  **R** CTC TGG TGG GGG TGT CTG GTT CA | 316 bp, 59°C |
| trkA NM_001033124 | **F** TCT CTG CCG CCC TCT TCC TTT CTG  **R** TCA AGT GGG AGC TAG GGG AGG GAG | 282 bp, 59°C |
| TRKB NM_001025074 | **F** TCC AAG TTT GGC ATG AAA GGC CCA  **R** TGG GTC GCC CTC CAC ACA GAC | 369 bp, 65°C |
| PERIPHERIN  NM_013639 | **F** ACC CGG GAT GGG GAG AAG GTG  **R** TGG ATC AGG CTG GCG CTT GC | 214 bp, 58°C |
| B3gat1  NM_029792 | **F** TGC TTG CTG TGC ACA AGG ATG AG  **R** GCG AGG GTC TCG GGC ATC AC | 392 bp, 64°C |
| HNK1-ST  NM_145142 | **F** CCT CCA AAC CCA GGC CGC AG  **R** GCA TCT TCC CAG TTG GCT TCG GG | 292 bp, 60°C |
| SCN1A NM_018733 | **F** CAG CAG GCA GCG GCT ACA AC  **R** GGG TGG ACA TTT CTG CCT G | 834 bp, 63°C |
| SCN2A AJ810516.1 | **F** GTT TCA GCA GAT GCT GGA GC  **R** GCA TGG AGG GCA TTT CTG TC | 853 bp, 55°C |
| SCN3A NM_018732 | **F** CAG CAG ATG TTG GAG CAG TTG  **R** CAG CAT GGT GGA CAC TTC TG | 707 bp, 55°C |
| SCN4A NM_133199 | **F** GG AGT TCC AAC AGA TGC TTG  **R** GTG TGC ACA CTT GTA CCA CC | 262 bp, 50°C |
| SCN5A NM_021544 | **F** CAG GAG GCC ATG GAG ATG CTC  **R** GAT TCC AGC ATG GTG GAC AC | 763 bp, 55°C |
| SCN7A NM_009135 | **F** GTG AAA GAA CTC GAC GAA G  **R** GGG CAT TTC TTC CTG GAT GT | 161 bp, 50°C |
| SCN8A, NM_001077499 | **F** CAG AAA TGT CGC TGC CGA GAC AG  **R** CCA GGT ACT TGG ATG CCA AG | 898 bp, 55°C |
| SCN9A NM_018852 | **F** CGA GGA ACA GAA CCA GGC CAA C  **R** GCT TGC TCT GCT CAT GGC CC | 865 bp, 58°C |
| SCN10A NM_001205321 | **F** GCT GGT CAT CTT CCT TGG ATC ATT  **R** GAA TTT CTT CCA CTT GGG GCA GCA | 828 bp, 58°C |
| SCN11A NM_011887 | **F** CAG AAA TGT CGC TGC CGA GAC AG  **R** CCA GGT ACT TGG ATG CCA AG | 446 bp, 55°C |
